# Supplementary material for: The Weight-Based Feature Selection (WBFS) Algorithm Classifies Lung Cancer Subtypes Using Proteomic Data
Source: Entropy (Basel). 2023 Jun 29;25(7):1003. doi: 10.3390/e25071003 (PMC10378569; doi:10.3390/e25071003)
Supplement: Supplementary file 1 [file entropy-25-01003-s001.zip › entropy-2345429-supplementary.pdf]

Table S1. Multiple regression results of the selected features in disease classification

**Variable**	**Coefficient**	**Std. Error**	**t**	***P* value**
Constant	0.3944			
BRD4	0.08126	0.02732	2.975	0.0030
CD26	-0.3421	0.04634	-7.382	<0.0001
DUSP4	-0.1118	0.01843	-6.067	<0.0001
GAPDH	0.03506	0.01002	3.498	0.0005
INPP4B	-0.09185	0.01605	-5.723	<0.0001
MIG6	-0.2995	0.04642	-6.452	<0.0001
NDRG1_pT346	0.06812	0.01274	5.346	<0.0001
TFRC	0.1152	0.01546	7.449	<0.0001

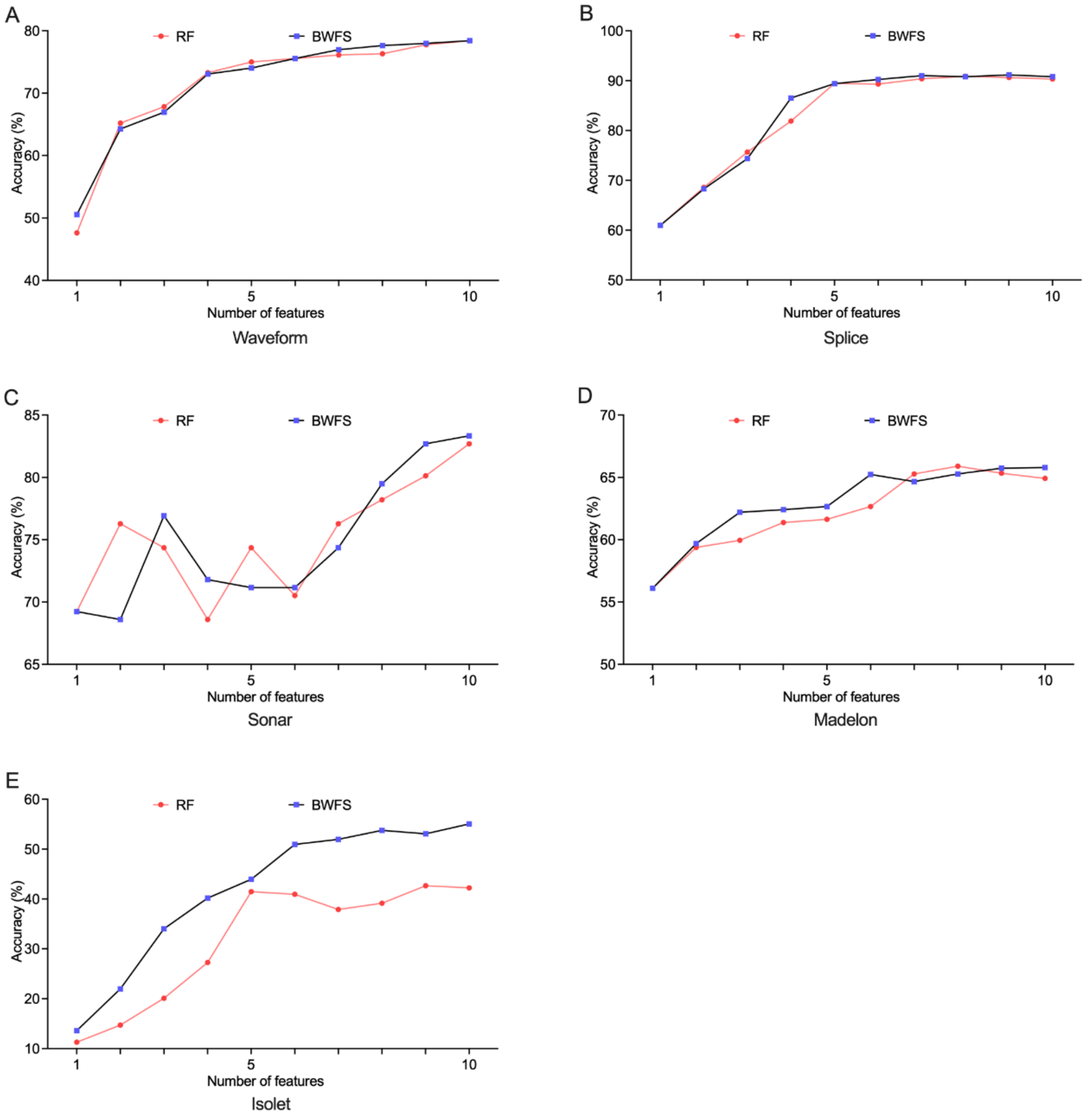

Figure S1. BWFS vs. RF classification accuracy comparison based on five datasets: (A) Waveform, (B) Splice, (C) Sonar, (D) Madelon, (E) Isolet. The X axis shows the number of selected features, and the Y axis shows the average classification accuracy of three classifiers (KNN, NBC and LibSVM).
